# The Impact of Early Versus Late Platelet and Neutrophil Recovery after Induction Chemotherapy on Survival Outcomes of Patients with Acute Myeloid Leukemia

**DOI:** 10.4274/tjh.galenos.2019.2019.0154

**Published:** 2020-05-06

**Authors:** Rafiye Çiftçiler, İbrahim C. Haznedaroğlu, Nilgün Sayınalp, Osman Özcebe, Salih Aksu, Haluk Demiroğlu, Hakan Göker, Ümit Yavuz Malkan, Yahya Büyükaşık

**Affiliations:** 1 Hacettepe University Faculty of Medicine, Department of Hematology, Ankara, Turkey; 2 Dışkapı Training and Research Hospital, Department of Hematology, Ankara, Turkey

**Keywords:** Acute myeloid leukemia, Platelet recovery, Neutrophil recovery

## Abstract

**Objective:**

The prognosis of patients with acute myeloid leukemia (AML) is affected by factors that are both patient- and disease-specific. The aim of this study is to evaluate the impact of early versus late platelet and neutrophil recovery after induction chemotherapy on survival outcomes of AML patients.

**Materials and Methods:**

A total of 181 patients with AML who were treated in our tertiary center between 2001 and 2018 were evaluated. Neutrophil and platelet recovery times were accepted as the periods from the beginning of induction chemotherapy to a neutrophil count of ≥0.5x10^9^/L and a platelet count of ≥20x10^9^/L 3 days in a row, respectively. The median time to platelet recovery was 25 days (range=12-52) for all patients. Therefore, platelet recovery in the first 25 days was defined as early platelet recovery (EPR) and at ≥26 days it was defined as late platelet recovery (LPR). The median time to neutrophil recovery was 28 days (range=13-51) for all patients. Therefore, neutrophil recovery in the first 28 days was defined as early neutrophil recovery, and at ≥29 days it was defined as late neutrophil recovery.

**Results:**

The 5-year overall survival (OS) rates for patients who had EPR and LPR after induction chemotherapy were 62% and 23%, respectively (p<0.001). The 5-year disease-free survival (DFS) rates for patients who had EPR and LPR after induction chemotherapy were 57% and 15%, respectively (p<0.001).

**Conclusion:**

Short bone marrow recovery time may indicate better healthy hematopoiesis and marrow capacity associated with longer OS and DFS.

## Abstract (tr)

**Amaç:**

Akut myeloid lösemili (AML) hastaların prognozu, hem hastaya hem de hastalığa özgü faktörlerden etkilenmektedir. Bu çalışmanın amacı, indüksiyon kemoterapisi sonrası erken ve geç trombosit ve nötrofil iyileşmesinin akut myeloid lösemi hastalarının sağkalım sonuçları üzerindeki etkisini değerlendirmektir.

**Gereç ve Yöntemler:**

2001-2018 yılları arasında üçüncü basamak sağlık merkezimizde tedavi edilen 181 AML hastası çalışmaya alındı. Nötrofil ve trombosit iyileşme süreleri, indüksiyon kemoterapisinin başlangıcından itibaren sırasıyla 3 gün süreyle nötrofil sayısının 0,5×10^9^/L’ye ve trombosit sayısının 20×10^9^/L’ye ulaşması olarak kabul edildi. Trombosit iyileşmesinin ortalama süresi tüm hastalar için 25 gündü (12-52). Bu nedenle, ilk 25 günde trombosit iyileşmesi erken trombosit iyileşmesi ve ≥26 gün geç trombosit iyileşmesi olarak tanımlandı. Nötrofil iyileşmesine kadar geçen ortalama süre tüm hastalar için 28 gündür (13-51). Bu nedenle, ilk 28 günde nötrofil iyileşmesi erken nötrofil iyileşmesi ve ≥29 gün geç nötrofil iyileşmesi olarak tanımlandı.

**Bulgular:**

İndüksiyon kemoterapisi sonrası erken ve geç trombosit iyileşmesi olan hastalar için 5 yıllık genel sağkalım sırasıyla %62 ve %23 olarak saptandı (p<0,001). İndüksiyon kemoterapisi sonrası erken ve geç trombosit iyileşmesi olan hastalar için 5 yıllık hastalıksız sağkalım sırasıyla %57 ve %15 saptandı (p<0,001).

**Sonuç:**

Sonuç olarak, kısa kemik iliği iyileşme süresi, daha uzun genel ve hastalıksız sağkalım ile ilişkili daha sağlıklı bir hematopoez/kemik iliği kapasitesini gösterebilir.

## Introduction

The clinical outcome of patients with acute myeloid leukemia (AML) varies across a wide spectrum, ranging from survival of a few days to remission. Therefore, the prediction of outcome is vital for those patients [1]. Prognosis of patients with AML is affected by factors that are both patient- and disease-specific. The most significant disease-specific prognostic factors at the time of diagnosis of AML are cytogenetics and molecular abnormalities [2]. On the other hand, the most important patient-specific prognostic factor is age at diagnosis [3]. Estimating resistance to treatment in patients with AML is extremely important for critical therapeutic decisions and follow-up of the patient [4]. Very limited data are available regarding the association between AML prognosis and bone marrow recovery kinetics following induction chemotherapy [5,6,7]. The aim of this study was to evaluate the impact of early versus late platelet and neutrophil recovery after induction chemotherapy on the survival outcomes of AML patients.

## Materials and Methods

### Study Design and Data Collection

This study was performed in a retrospective manner. All clinical data were collected from hospital medical records. As a result of the application standards of the hospitals of Hacettepe Medical School, it has been recognized from the patient records that all of the studied patients had given informed consent at the time of hospitalization and before the administration of chemotherapy and other relevant diagnostic/therapeutic standards of care.

### Patient and Disease Characteristics

Neutrophil recovery time (NRT) and platelet recovery time (PRT) were accepted as the periods from the beginning of induction chemotherapy to a neutrophil count of  ≥0.5x10^9^/L 3 days in a row and a platelet count of ≥20×10^9^/L 3 days in a row (without transfusion support), respectively. The median time to platelet recovery was 25 days (range=12-52) for all patients. Therefore, platelet recovery in the first 25 days was defined as early platelet recovery (EPR) and at ≥26 days it was defined as late platelet recovery (LPR). The median time to neutrophil recovery was 28 days (range=13-51) for all patients. Therefore, neutrophil recovery in the first 28 days was defined as early neutrophil recovery (ENR) and at ≥29 days it was defined as late neutrophil recovery (LNR).

In this study, patient inclusion criteria were as follows: age >18 years at the time of diagnosis, patients who received first induction chemotherapy, and achievement of complete remission after induction chemotherapy. Patients with refractory AML and patients who were diagnosed with acute promyelocytic leukemia were not included in this study. All patients included in the study received idarubicin (12 mg/m^2^ IV push on each of the first 3 days of treatment) and Ara-C (100 mg/m^2^ daily as a continuous infusion for 7 days) as induction chemotherapy [8].

### Statistical Analyses

Statistical analyses were performed using SPSS 25 (IBM Corp., Armonk, NY, USA). The variables were investigated using visual (histograms, probability plots) and analytical methods (Kolmogorov-Smirnov/Shapiro-Wilk tests) to determine whether they were normally distributed or not. Statistical comparisons were made using chi-square tests for categorical data. The Student t-test for two independent samples was used for comparison of continuous numerical data. Survival analyses were made using Kaplan-Meier tests. Multivariate analysis of predictors of survival was performed using the Cox regression test. Parameters with p≤0.10 in univariate tests were included in the multivariate analysis, while p<0.05 was considered to indicate statistical significance.

## Results

### Patients’ Characteristics

A total of 450 AML patients admitted to our hospital between 2001 and 2018 were screened for this study. Patients with refractory AML, patients who did not achieve complete remission after the first induction chemotherapy, and patients who died during induction chemotherapy were not included in the study. Patient characteristics are summarized in Table 1. There were 106 (57.9%) males and 77 (42.1%) females with a median age of 44 (range=18-69) years at diagnosis. Karyotype analyses were available for 159 patients: 6 patients (3.7%) were in the favorable-risk group, 101 (63.5%) patients were in the intermediate-risk group, and 54 (33.9%) patients were in the adverse-risk group according to the European LeukemiaNet classification [9]. The number of patients classified as having Eastern Cooperative Oncology Group performance status (ECOG PS) 0, 1, 2, and 3 were 4 (2.2%), 87 (48.1%), 78 (43.1%), and 12 (6.6%), respectively [10]. According to periods, LPR was seen in fewer patients between 2011 and 2018 than in 2001-2010 (p=0.01). Preexisting myelodysplastic syndrome or secondary AML was seen more in patients with LPR than in patients with EPR (p=0.02).

There were no statistically significant differences between the two groups in terms of median age (p=0.10), sex (p=0.18), cytogenetic risk group (p=0.77), and ECOG PS (p=0.06). Mortality (66.3% vs. 30.4%, p<0.001) and relapse rate (47.2% vs. 29.3% p=0.01) were higher in patients who had LPR than EPR after induction chemotherapy. Nonrelapse mortality rate (NMR) was higher in patients who had LPR than EPR (28.1% vs. 9.8%, p=0.001). Major causes of NRM were infections (20 vs. 8), heart attack (3 vs. 0), acute renal failure (1 vs. 0), and graft-versus-host disease (1 vs. 0) in LPR and EPR patients, respectively.

### Overall Outcomes

Median follow-up time was 21 months (range=1.5-220) for all patients. The 3-year overall survival (OS) rates for patients who had EPR and LPR were 68% and 40%, respectively. The 5-year OS rates for patients who had EPR and LPR were 62% and 23%, respectively (p<0.001). The 3-year disease-free survival (DFS) rates for patients who had EPR and LPR were 64% and 28%, respectively. The 5-year DFS rates for patients who had EPR and LPR were 57% and 15%, respectively (p<0.001).

**Figure 1:**
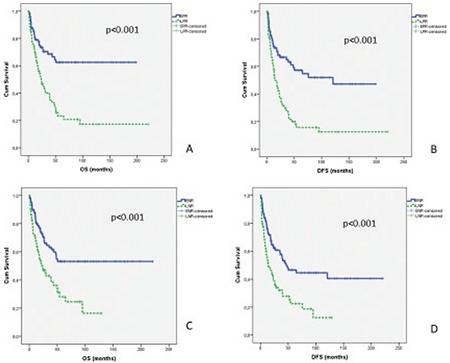
Overall survival (OS) and disease-free survival (DFS) of patients (A-B for EPR and LPR groups, C-D for ENR and LNR groups).

The 3-year OS rates for patients who had ENR and LNR were 63% and 42%, respectively. The 5-year OS rates for patients who had ENR and LNR were 53% and 28%, respectively (p<0.001). The 3-year DFS rates for patients who had ENR and LNR were 57% and 32%, respectively. The 5-year DFS rates for patients who had ENR and LNR were 46% and 22%, respectively (p<0.001) (Figure 1).

### Cox Regression Analyses

In univariate analyses, factors affecting OS were age (p=0.004), cytogenetics (p<0.001), ECOG PS (p<0.001), ENR (p<0.001), and EPR (p<0.001) of the patients, as shown in Table 2. Cox regression analysis revealed the parameters predicting OS as cytogenetics (p<0.001), ECOG PS (p<0.001), and EPR (p=0.02) of the patients.

In univariate analyses, factors affecting DFS were age (p=0.006), sex (p=0.06), cytogenetics (p<0.001), ECOG PS (p<0.001), ENR (p=0.009), and EPR (p=0.001) of the patients. Cox regression analysis revealed the parameters predicting DFS as sex (p=0.002), cytogenetics (p<0.001), ECOG PS (p<0.001), and EPR (p=0.01) of the patients.

## Discussion

After induction chemotherapy, the duration of neutropenia and thrombocytopenia carries a risk of complications in AML patients. Some patients die from infections during the neutropenic period. Intracranial hemorrhage may be seen because of thrombocytopenia as a serious life-threatening complication. In this study, EPR was one of the significant independent parameters in multivariate analysis that included classical prognostic risk factors for OS and DFS. Since hematopoietic growth factors were used for neutrophil recovery in some patients, ENR may not have significantly resulted in long OS and DFS in multivariate analysis. Bone marrow reserve may be considered to be better in patients who had EPR and ENR. Patients with LPR and LNR may be considered more at-risk and donor screening may be initiated at an early stage for allogeneic hematopoietic stem cell transplantation (allo-HSCT).

AML prognosis is related to bone marrow recovery, cellular kinetics [5], and blast clearance after induction chemotherapy [11,12]. Some studies reported that an early response to induction chemotherapy was a strong and independent prognostic factor for survival in patients with de novo and relapsed AML [13,14,15]. Yamazaki et al. [16] showed that the regeneration of hematopoiesis after induction chemotherapy, and especially the recovery of platelets, is an important positive predictor for DFS in patients with AML. On the other hand, a previous study evaluated the survival outcomes of patients who underwent allo-HSCT with incomplete remission (CRi, bone marrow CR with absolute neutrophil count of <1,000/mm^3 ^and/or platelet count of <100,000/mm^3^) and complete remission (CR, bone marrow CR with absolute neutrophil count of ≥1,000/mm^3 ^and platelet count of ≥100,000/mm^3^). The study showed equivalent posttransplant outcomes between patients who were in CR and in CRi before allo-HSCT. Therefore, allo-HSCT can eliminate the negative effect of pretransplant blood count levels [17]. The major cause of NRM was infection; therefore, allo-HSCT might be considered in the nadir period for AML patients. However, it will be difficult to find a donor in such a short period and prepare the patient for allo-HSCT.

## Conclusion

Early bone marrow recovery may indicate a better healthy hematopoiesis and marrow capacity associated with longer OS and DFS. As PRT and NRT are very easy to detect, they can be used as prognostic indicators in countries with limited laboratory facilities. Our results support the impression that an accelerated platelet and neutrophil recovery following chemotherapy could be accepted as a promising sign of good prognosis and thus good future response to therapy in AML. The results of this study are important for prediction of the prognosis of newly diagnosed AML patients.

## Figures and Tables

**Table 1:**
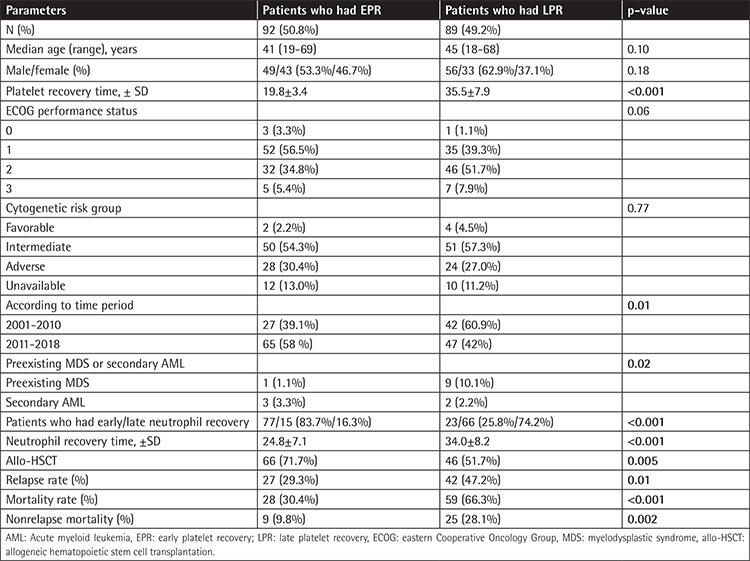
Baseline characteristics of AML patients.

**Table 2:**
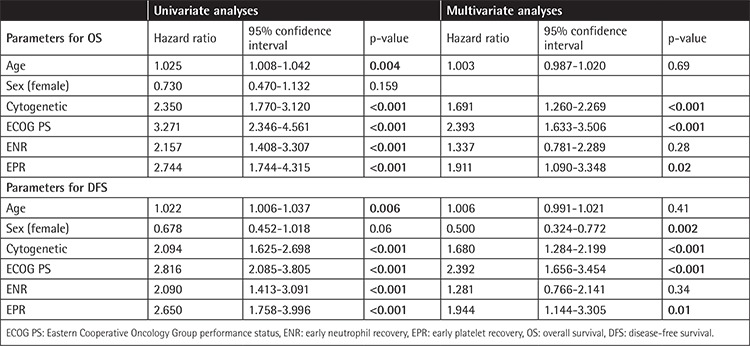
Univariate and multivariate analyses of overall survival (OS) and disease-free survival (DFS).
